# SARS-CoV-2 associated Guillain–Barré syndrome

**DOI:** 10.1007/s00415-020-10133-w

**Published:** 2020-08-08

**Authors:** Teodor Pelea, Ursula Reuter, Christine Schmidt, Raimondo Laubinger, Robert Siegmund, Bjoern Wito Walther

**Affiliations:** 1 Department of Neurology, Care Medicine, SRH Zentralklinikum Suhl, Albert-Schweitzer-Strasse 2, 98527 Suhl, Germany; 2 Department of Anesthesiology and Intensive, Care Medicine, SRH Zentralklinikum Suhl, Suhl, Germany; 3 Community Laboratory, Suhl, Germany

**Keywords:** Guillain–Barré syndrome, COVID-19, SARS-CoV-2, Bilateral facial diplegia

## Abstract

Presented herein is a severe case of SARS-CoV-2 associated Guillain–Barré syndrome (GBS), showing only slight improvement despite adequate therapy. To date, only few cases of GBS associated with this infection have been described. This case report summarizes the insights gain so far to GBS with this antecedent trigger. So far, attention has mostly focused on complications of the CNS involvement. Taking into account that GBS can cause a considerable impairment of the respiratory system, clinicians dealing with SARS-CoV-2 positive-tested patients should pay attention to symptoms of the peripheral nervous system. As far as we know from this reported case and the review of the current literature, there seems to be no association with antiganglioside antibodies or a positive SARS-CoV-2 RT-PCR in CSF. An obvious frequent occurrence of a bilateral facial weakness or bilateral peripheral facial diplegia should be emphasized.

## Introduction

Neurological symptoms associated with coronavirus (CoV) studies have shown that these viruses have neuro-invasive and neurotrophic characteristics [1]. The infections with CoV can affect the nervous system [1]. 36.4% among 214 hospitalized patients infected with COVID-19 have reported neurological symptoms.[2]. The authors describe nervous system-associated symptoms as including dizziness, headache, hypogeusia, hyposmia, muscle damage, and ischemic and hemorrhagic stroke [2]. The current hypothesis is that CoV, together with the host immune mechanisms, may turn these infections into persistent infections that affect also neurological structures. First of all, central nervous system (CNS) involvement is assumed. Pathogenesis of nervous system injury caused by CoV includes acute cerebrovascular diseases, toxic encephalopathy and viral encephalitis [1]. The peripheral nervous system seems not to be affected by a direct virus-mediated pathway. GBS is an acute immune-mediated disease of the peripheral nerves and nerve roots that is usually elicited by various infections [3]. The diagnosis should be based on the diagnostic criteria of Asbury and Cornblath [4]. Respiratory tract or gastrointestinal infections, up to 2–6 weeks prior to the onset of neurological symptoms of GBS, have been reported by 50–70% of the affected patients [3, 5]. CoV infections can cause multiple systemic infections. Respiratory complications are the most recognizable symptoms, similar to severe acute respiratory syndrome coronavirus (SARS-CoV). Pulmonary disorder and respiratory insufficiency are the main problems linked to the actual present pandemic, SARS-CoV-2 infection [6]. After an incubation period of approximately 5.2 days, the prevailing symptoms include fever, cough, dyspnea, myalgia, headache, and diarrhea [6]. Therefore patients with SARS-CoV-2 infection are at risk of being affected by coincident immune-mediated neurological diseases such as GBS.

### Case report

A 56-year-old Caucasian woman with a medical history of mild arterial hypertension (valsartan 40 mg) and hypothyreosis (l-thyroxin 25 µg) suffers from a dry cough, mild fever and a general weakness. In the context of the COVID-19 pandemic, SARS-CoV-2 RT-PCR on nasopharyngeal swab was performed and tested positive. A quarantine at home was decreed. The presumed contact to an infected person has been 12 days before the first symptoms appeared. Seven days later, she noticed weakness of her limbs while climbing stairs and a tingling sensation in all fingertips and toes. She was admitted to our emergency department 3 days after the occurrence of these neurological symptoms. On physical examination, the patient was afebrile with blood pressure at 135/82 mmHg, heart rate of 110 beats/min, respiratory rate at 18/min, and oxygen saturation of 95% on room air. She was conscious and had no dyspnea at the time of hospitalization. The neurological examination showed no meningeal irritation signs or abnormalities in the cranial nerve status. The muscle strength examination showed paresis in four limbs with a Medical Research Council (MRC) scale of 4/5 in the proximal, 3/5 in the distal upper extremities, 4/5 in the proximal, and 3/5 in distal in the lower extremities. Deep tendon reflexes were generally absent and there were no signs of upper motor neuron disorder. There was a reduction in the vibration of the knees from 2/8 in the 128 Hz tuning fork test, and fine touch sensation was bilateral stocking shaped. There was no spine sensory level. Meningeal irritation signs and upper motor neuron disorder signs were negative.

The laboratory results were as follows: white blood cell count 11,400 cells per microliter (neutrophils = 82.7%; lymphocytes = 10.4%), fibrinogen 4.93 g/l, C-reactive protein < 2 mg/l, hemoglobin 7.8 g/dL, serum glucose 5.79 mmol/l, and further normal results for blood urea nitrogen, creatinine, ALAT, ASAT, LDH, GGT, sodium, potassium, INR, PTT, IgG, IgA, IgM, and complete urinalysis. Anti-ganglioside antibodies (GM1-, GQ1b-antibodies) were absent.

The analysis of cerebrospinal fluid (CSF) showed a cell count of 9 Mpt/l (lymphocytes and monocytes), protein of 0.575 g/l, glucose 3.74 mmol/l and lactate 2.2 mmol/l, and no oligoclonal bands. The SARS-CoV-2 RT-PCR in CSF was performed and tested negative. Biological tests were not in favor of a recent infection with *Borrelia, Treponema pallidum, Campylobacter jejuni*, mycoplasma, EBV, HSV1 or 2, and hepatitis E.

A CT scan of the brain and MRI of the spine showed no abnormalities. Lung CT at admission showed leaky infiltrates in the right lower lobe, at the tip and dorsally; infiltrates most likely began in the dorsal left in the lower lobe, increased, with maximum 22 mm paratracheal and infracarinal lymph nodes.

The patient was admitted to ICU and further treatment was carried out in strict compliance with the isolation measures. Our patient received PPh every 2 days, and there was a clinical deterioration in spite of this treatment during the first 5 days. The patient developed a flaccid, severe tetraparesis of 3/5 in the proximal, 1/5 in the distal of the upper extremities and 3/5 in the proximal and 0/5 in the distal of the lower extremities for dorsal extension, 2/5 for flexion, a trunk instability, and also bilateral peripheral facial nerve palsy (House–Brackmann grade 5). There were autonomous symptoms with a tachycardic heart action until 120/min and a severe orthostatic dysregulation, with no further possibility of sitting upright. She showed a tendency for clinical improvement after the third course of PPh. Seven courses of PPh were performed. The PPh caused a slightly further clinical improvement with asymmetrical improvement of facial paresis and tetraparesis, but a clinical stagnation of the improvement during the following 5 days. The patient was still unable to sit upright because of orthostatic collapsing and trunk instability. Therefore, we added 5 days after the last PPh 0.40 g/kg/day intravenous immune globulins for a duration of 5 days.

We performed the neurophysiological study and nerve sonography only on day 10 according to the isolation requirements. Nerve sonography, as a painless technique for bedside-imaging nerve pathology, demonstrated a hypoechoic ultrasonographic cervical spinal nerve enlargement. The cross-sectional area of the C6 root was measured as 21 mm^2^ and C7 root as 22 mm^2^. Enlarged cervical spinal and peripheral nerves detected by ultrasound were identified as an early marker for Guillain–Barré syndrome [7].

Electroneurographic parameters demonstrate the typical delay of distal motor latency, and F-wave latency and decrease of conduction velocity, as well as decreased amplitudes at compound muscle action potential. There was mild decrease of conduction velocity of sensory nerve action potential changes at the arm nerves. The findings are basically consistent with acute motor accented and axonal demyelinating neuropathy (Table 1).

**Table 1:** Nerve conduction study parameters day 10

Nerve stimulated	Stimulation SITE	Amplitude (m in mV; s in µV)			Latency (ms)			Conduction velocity (m/s)			F-Wave (ms)/F-wavepersistence (%)		
		Right	Left	Norm	Right	Left	Norm	Right	Left	Norm	right	left	Norm (height 175 cm)
Medianus (s)	Wrist	8.9	17.2	≥ 6	3.7	4.25	3.2	35	33	≥ 44			
Ulnaris (s)	Wrist	10	31	≥ 5	2.7	3.15	2.8	40	35	≥ 44			
Suralis (s)	Calf	9	9	≥ 5	2	2.1	2	49	50	≥ 40			
Medianus (m)	Wrist	5	5.6	≥ 3.5	6.6	6.2	≤ 4.2				39.7/80	33.6/30	≤ 31/50–100
	Antecubital fossa	4.2	5.6	≥ 3.5	12.1	10.9		44	45	≥ 50			
Ulnaris (m)	Wrist	0.7	1.8	≥ 2.8	4.6	4	≤ 3.4				35	33	≤ 31/50–100
	Below elbow	1.2	0.7	≥ 2.8	11	11.2		39	35.9	≥ 48			
Tibialis (m)	Ankle	1.5	0.9	≥ 2.9	4.9	7.9	≤ 6				nr	73/20	≤ 58/50–100
	Popliteal fossa	0.5	0.3	≥ 2.9	16.4	19.8		33	33	≥ 41			
Peroneus (m)	Ankle	2.2	2.1	≥ 2.5	3.5	5.1	≤ 5				70/30	66/20	≤ 57/50–100
	Fibula	0.57	0.69	≥ 2.5	14.1	15		30	33	≥ 40			

*nr* no response

There was no fever or respiratory complaints over the time. Further treatment was given in the intermediate care unit, but there was only a slight clinical improvement over the next few days. The clinical course up to the time of transfer to a rehabilitation facility and the eletroneurographic findings with evidence of an axonal motor damage can indicate a complicated course with a prolonged and possible defective healing.

## Discussion

Only one case series [8] and a few case reports [9, 11] show an association between SARS-CoV-2 infection and GBS. The presented well-documented case report shows all characteristics of a typical, but severe, course of GBS. The association with the SARS-CoV-2 infection in the present case is without a doubt because of the strict time connection. The clinical course regarding the COVID 19 disease and the respiratory symptoms was uncomplicated. The main complaint was the neurological complication with GBS. Severe course of GBS-associated SARS-CoV-2 infections occur also in patients with mild respiratory symptoms, but must be taken into account with seriously ill cases. With COVID-19 disease due to a general impairment, the neurological symptoms can be easily overlooked. Since GBS can cause or exacerbate respiratory symptoms, it should take into account the suspect courses of COVID 19. It would be helpful if clinical, paraclinical, or electrophysiological findings were found that would facilitate the diagnosis of GBS. To date, the previously described courses of the SARS-CoV-2 infection-associated GBS do not describe a special clinical pattern. To date, available references summarizing the following points include a total of nine published cases.

A remarkable clinical pattern in our case was that there was bilateral peripheral facial nerve palsy. This clinical symptom has been reported in one other case report [10] and 3/5 cases in the Italian series reported a facial diplegia in one case and facial weakness in two cases [8]. Therefore, we can describe a bilateral facial involvement in five out of nine patients (55.5%) and a documented bilateral facial diplegia in 3/9 patients (33,3%). Facial nerve involvement in GBS is a common finding in 27–50% [12]. There are no data available for a bilateral seventh nerve involvement in GBS. Estimated data reported up to 12–25% [11].

The CSF parameters show no specific pattern. The SARS-CoV-2 RT-PCR in CSF was performed in our patient and in the Italian series of five patients [8] and was negative in all cases.

Antiganglioside antibodies (GM 1-, GQ1b-antibodies) may indicate special GBS subtypes. They were analyzed in our case and three out of five in the Italian series [8] tested negative.

Nerve conduction studies have been performed in our case and two other case reports [9, 10]. An axonal affection pattern is reported in two out of three cases. Except for the presented case, the clinical course of the other cases is not well documented. So the data do not allow a discussion over a prognostic value of the present electrophysiological data.

So far, attention has mostly focused on complications of the CNS involvement. Taking into account that GBS can cause a considerable impairment of the respiratory system, clinicians dealing with SARS-CoV-2 positive-tested patients should have to pay attention to symptoms of the peripheral nervous system. As far as we know from these few reported cases, there seems to be no association with antiganglioside antibodies or a positive SARS-CoV-2 RT-PCR in CSF. The occurrence of a bilateral facial weakness or bilateral peripheral facial diplegia should be emphasized. This finding and the appearance of specific electrophysiological pattern should be shown in further investigations.
